# Constrained Dual Scaling for Detecting Response Styles in Categorical Data

**DOI:** 10.1007/s11336-015-9458-9

**Published:** 2015-04-08

**Authors:** Pieter C. Schoonees, Michel van de Velden, Patrick J. F. Groenen

**Affiliations:** Econometric Institute, Erasmus University Rotterdam, Rotterdam, The Netherlands

**Keywords:** response style, dual scaling, correspondence analysis, splines, nonnegative least squares, $K$-means

## Abstract

Dual scaling (DS) is a multivariate exploratory method equivalent to correspondence analysis when analysing contingency tables. However, for the analysis of rating data, different proposals appear in the DS and correspondence analysis literature. It is shown here that a peculiarity of the DS method can be exploited to detect differences in response styles. Response styles occur when respondents use rating scales differently for reasons not related to the questions, often biasing results. A spline-based constrained version of DS is devised which can detect the presence of four prominent types of response styles, and is extended to allow for multiple response styles. An alternating nonnegative least squares algorithm is devised for estimating the parameters. The new method is appraised both by simulation studies and an empirical application.

## Introduction

A major issue in questionnaire-based research is the presence of response styles. A response style, sometimes also known as response bias or scale usage heterogeneity, can be described as systematic bias due to a respondent’s tendency to respond to survey items regardless of its content (Van Rosmalen, Van Herk, & Groenen, 2010). Paraphrasing, a response style is the manner in which a person uses a rating scale, an example being extreme response style where the respondent, *for no substantial reason,* prefers to use the endpoints of the Likert scale more often than the intermediate rating categories.

Response styles can invalidate statistical analyses since they are completely confounded with the substantial information contained in the data and hence biases results in nontrivial ways (Baumgartner & Steenkamp, 2001). The problem manifests itself when different respondents resort to different response styles within the same data set. Advanced methods, such as the latent-class multinomial logit model of Van Rosmalen et al. (2010), the multidimensional ordinal IRT model of De Jong and Steenkamp (2010), or the ordinal regression model with heterogeneous thresholds of Johnson (2003), have been developed to deal with the data analysis when response style contamination is relevant. None of these appear to have achieved much popularity in practice.

Existing models often require a substantial investment of resources for its implementation, estimation and/or interpretation. As an alternative, the method presented in this paper results in a data set cleaned of the effects of response styles so that any analyses appropriate for the continuous nature of this cleaned data can be conducted. Furthermore, this method has three additional purposes, namely to (i) determine whether different response styles are present in categorical data; (ii) identify the respondents associated with each response style; and to (iii) classify the identified response styles into four different types. Software which implements the method in the R software environment (R Core Team, 2014) is available from the first author.

The proposed method is a variant of dual scaling (DS) for rating data (Nishisato, 1980a), also referred to as successive categories data in the DS literature. DS is an exploratory multivariate method, akin to correspondence analysis or CA (e.g. Greenacre, 2007). In the special case of rating data, DS however differs from CA in a manner that implicitly caters for response styles by including parameters for the Likert scale categories in an innovative way. These parameters allow for the detection of frequent (or infrequent) usage of certain ratings since the optimal scores assigned by DS to these parameters depend on how often each rating occurs in the data. The new method builds on this aspect of DS by including monotone spline functions to model the response styles and by allowing for multiple response styles through latent classes.

The literature on response styles (also known as scale-usage bias or heterogeneity) can be traced back at least to the work of Cronbach in the 1940s (e.g. Cronbach, 1941, 1942, 1946, 1950). For an overview of the early work, see for example Rorer (1965). A more recent set of references can be found in Baumgartner and Steenkamp (2001). Krosnick (1999) discusses the origins of response styles as a shift in the procedure whereby a response is formulated; this is also known as satisficing in the literature (e.g. Krosnick, 1991). The use of so-called personal equations with double coding, as known in the French school of CA, is a related method of dealing with differences in the interpretation of rating scales at the respondent level (e.g. Benzécri, 1992; Murtagh, 2005).

The next section focuses on a closer discussion of response styles. Section 3 introduces spline functions for modelling response styles, explains the new methodology and details an alternating least squares (ALS) algorithm for solving an extended version of the DS problem. A simulation study is conducted in Section 4 to assess the strengths and weaknesses of the method. Finally, an application (Section 5) is presented.

## Overview of Response Styles

It is assumed that the process of formulating a response to a survey item requires the respondent to map a latent opinion, preference or some similar concept to a Likert scale. For example, the respondent may be asked how much she agrees with a certain statement using a scale with categories ranging from “1—Totally Disagree” to “5—Totally Agree.” During the cognitive process of formulating the answer, the respondent first forms an opinion about the survey item and subsequently needs to decide how to transform or map this opinion to the presented rating scale (see for example Weijters and Baumgartner (2012)). The mathematical properties of this response mapping from the latent to the Likert scale determines whether a respondent exhibits a response style or not.

Specifically, a response style can be defined as a monotone nonlinear response mapping (Van de Velden, 2007). If this transformation is linear, no response style is present. Consequently, once a method is available to estimate response mappings, the presence of response styles can be assessed by looking at the curvature properties of the estimated mappings. These steps are carried out in subsequent sections. In the case where Likert scales are used these transformations are step functions, but for the moment it is more intuitive to consider continuous transformations.

**Fig. 1:**
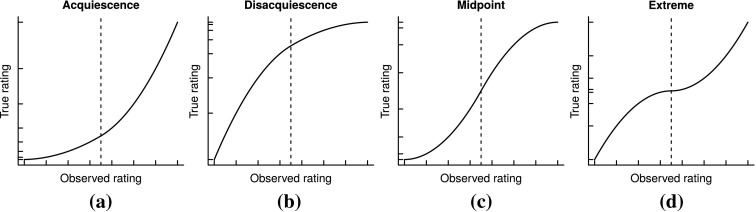
Examples of (inverse) response style functions mapping the true item content scale (*vertical axis*) into the observed measurement scale (*horizontal axis*).

Four different response styles are considered here, as depicted in Figure 1. This figure shows different possible inverse mappings from the rating supplied by the respondent on the horizontal axis to the respondent’s true latent opinion on the vertical axis. The inverse transformations are shown since these must be estimated from the observed data.

The different styles can be characterized by which parts of the latent opinion scale are stretched and which parts are shrunk. These are shown by the rug plots on the respected axes in Figure 1. For ease of exposition, it is assumed here that the true latent opinion comes from a uniform distribution. The rug on the horizontal axis partitions the axis into intervals of equal length, with each interval receiving a rating on the Likert scale. Here a seven-point scale is employed. The rug on the vertical axis shows the effect that the response style transformation has on the intervals of equal length. Hence these transformations characterize the following four response styles:

- *Acquiescence* (ARS) shrinks the lower part of the latent scale and stretches the upper part indicating that higher ratings are favoured (panel (a));
- *Disacquiescence* (DRS) in contrast favours lower ratings by stretching and shrinking the lower and upper parts of the latent scale, respectively (panel (b));
- *Midpoint responding* (MRS) reflects a tendency to frequent the middle categories of the rating scale (panel (c)); and
- *Extreme responding* (ERS) in contrast means that the endpoints of the rating scale is used more often than the middle categories (panel (d)).

A critical concept is that the boundaries dividing the latent preference scale into the different rating categories, that is the tick marks on the vertical axes in Figure 1, determines which response style is present. If these boundaries are equally spaced, no response style is present. Any significant deviations however give a cause to believe that a response style is present.

The methodology outlined in the next section makes use of these boundaries to provide an estimate of the response mappings of groups of individuals.

## Methodology

Consider the situation where a set of $m$ objects or survey items are being rated on a $q$-point Likert scale, enumerated as $1$ to $q$. Due to the ordinality, this is sometimes known as successive categories data (Nishisato, 1980b, 1994). It is supposed that $n$ individuals are asked to rate the objects according to their preference. Objects may receive equal ratings, and it is assumed that there exists a fixed but unknown preference structure for the set of objects, such as a population mean. Let $\mathbf {X}$ denote the $n \times m$ data matrix. Note that the method detailed below requires all items to use a common rating scale.

The next subsection discusses using DS for analysing successive categories data in general, making use of the method’s relationship with correspondence analysis. Monotone quadratic splines for modelling response styles are introduced in Section 3.2. Subsequently the DS method is modified to utilize these splines together with latent classes to allow for multiple response styles. An alternating nonnegative least squares algorithm is described for fitting the model in Section 3.4. Selecting the number of latent response style groups (Section 3.5) and creating a data set purged of the effects of response styles (Section 3.6) are also discussed.

### Dual Scaling of Successive Categories Data

Dual scaling is a multivariate exploratory statistical technique which is equivalent to correspondence analysis (CA) when analysing contingency tables (Van de Velden, 2000). For such cases, it is used to visualize departures from the independence assumption in the two-way contingency table in a low dimensional space, akin to principal components analysis (PCA) for continuous data (Nishisato, 1980a; Greenacre, 2007). However, for the successive categories data dealt with here there are important differences.

Both DS and CA deal with non-contingency table data by typically applying the standard procedure to a specific recoding of the data, designed to transform the data into a form that resembles a contingency table (Greenacre, 2007). This recoding requires the original data matrix $\mathbf {X}$ to be transformed before analysis, and for successive categories data in particular the recoding schemes differ in an important way. The usual CA method uses a doubling of columns (that is, adding an additional column to $\mathbf {X}$ for each object) to construct scales with “positive” and “negative” poles before applying ordinary CA (see Greenacre, 2007). However, Nishisato (1980b) proposes the following alternative method. This involves augmenting rating scale category thresholds or boundaries to the data, which increases the number of columns from $m$ to $m + q -1$, and then converting this to rank-orders. Although Nishisato’s original DS formulation focuses on a so-called dominance matrix (see Nishisato, 1980a), it has been shown that DS applied to these rank-orders are equivalent to doubling the rows (instead of the columns) of the matrix of rankings before applying CA (Van de Velden, 2000; Torres & Greenacre, 2002).

The method is perhaps best illustrated by an example. Consider transforming the following data matrix $\mathbf {X}$, where three objects $A$, $B$ and $C$ are rated by $n = 4$ respondents on a 5-point Likert scale (thus, $q = 5$). The first step requires augmenting 4 ($= q-1$) columns to $\mathbf X$, one column for each of the boundaries between the pairs of adjacent ratings. Let the boundaries be called $b_{1},\dots ,b_{4}$, where $b_{1}$ falls between ratings 1 and 2, and so forth up to $b_{4}$ which falls between categories 4 and 5. It suffices to assign scores midway between the rating categories to each boundary, to arrive at the augmented data matrix:1 Secondly, each row is converted to rankings, starting with a lowest rank of 0 and a highest rank of 6 ($= m + q - 2$) in this case. For ties the total ranking assigned to the tied objects is distributed equally. This yields the following result for the example:2Note that in general $\mathbf {T}$ has $n$ rows and $m + q - 1$ columns. DS also requires construction of the matrix $\mathbf {S}$ that would have resulted if $q$ was the lowest and not the highest rating of the Likert scale. This is easily achieved as

3 $$\begin{aligned} \mathbf {S} = (m + q - 2) \mathbf {1} \mathbf {1}^{'} - \mathbf {T}. \end{aligned}$$

Using the CA formulation of DS of Van de Velden (2000), a row-doubled ratings matrix $\mathbf {F}_{r}: 2n \times (m+q-1)$ is constructed as

4 $$\begin{aligned} \mathbf {F}_{r} = \begin{pmatrix} \mathbf {T} \\ \mathbf {S} \\ \end{pmatrix}. \end{aligned}$$

This matrix is subjected to CA, which assigns optimal scores in the vectors $\mathbf {a}$ and $\mathbf {b}$ to the rows and columns of $\mathbf {F}_{r}$, respectively. Since the aim is to assign to the boundaries ordered scores which are sensitive to rating scale use, a one-dimensional solution is used. This assignment is achieved by minimizing a least squares criterion $L(\mathbf {a}, \mathbf {b})$ through the singular value decomposition (Van de Velden, Groenen, & Poblome, 2009). In the present context $L$ is given by

5 $$\begin{aligned} L(\mathbf {a}, \mathbf {b} ) = c\Vert \mathbf {F}_{r} - \frac{1}{2}(m+q-2)(\mathbf {1} \mathbf {1}^{'} +\mathbf {ab}^{'})\Vert ^{2} \end{aligned}$$

where $c$ is a proportionality constant, $\mathbf {1}$ denotes vectors of ones of the appropriate lengths and $\frac{1}{2}(m+q-2)\mathbf {1} \mathbf {1}^{'}$ centres the rankings in $\mathbf {F}_{r}$. For identifiability, a constraint such as $\Vert \mathbf {a}\Vert = 1$ is imposed. The method is discussed in more detail in Section 3.3.

Note that an important consequence of the data recoding scheme is that the DS procedure provides coordinates for the boundaries. The effect of the boundaries is to retain the information on how different the original ratings assigned to the objects were before the rankings were constructed. The coding scheme also imposes ordinality on the object and the boundary scores in $\mathbf {b}$ by constructing rankings.

The optimal scores assigned to the boundaries can be used to detect response styles since they estimate the thresholds of the response mapping of the group of respondents, as was discussed in relation to Figure 1. Intuitively optimal scores assigned to the boundaries work as follows. If a specific rating category $j$ is used very often, the boundaries $b_{j-1}$ and $b_{j}$ will often receive rankings which differ substantially since the category is often filled. Consequently, the optimal scores assigned will differ significantly, indicating that respondents use the category very often. The same reasoning illustrates that when rating $j$ is used very infrequently, the optimal scores for $b_{j-1}$ and $b_{j}$ will be very similar. Therefore, when a group of respondents have the same response mapping, the method will be able to tell which type that mapping is.

In Section 3.3, latent classes will be introduced for the boundary scores which allows for multiple response styles. First, however, using monotone quadratic splines with the dual scaling method is discussed.

### Modelling Response Styles by Monotone Quadratic Splines

From Figure 1, it is evident that the four response styles considered can be completely described in terms of its curvature properties. By dividing the horizontal axes into two equal lower and upper parts, the four response styles are characterized by either concavity or convexity in the lower and upper parts of its domain. This is summarized in Table 1.

**Table 1:** Curvature properties of the four response styles.

Response style	Lower curvature	Upper curvature
No response style	None	None
Acquiescence	Convex	Convex
Disacquiescence	Concave	Concave
Extreme responding	Concave	Convex
Midpoint responding	Convex	Concave

For inferential and response style classification purposes, it will prove useful to parameterize the response style transformations considered here. Furthermore, using smooth functions will improve model parsimony and the stability of parameter estimation, as well as facilitate the process of purging the response styles from the data by interpolation (see Section 3.6). The family of monotone quadratic splines with a single interior knot is ideal for this purpose as it combines two quadratic polynomial functions in the adjacent intervals of the domain, subject to continuity and differentiability restrictions at the interior knot. These splines are either concave, convex or linear in the lower and upper halves of the domain and therefore reproduce all the curves described in Figure 1 and Table 1.

The splines have three non-constant basis functions (the so-called *I*-spline basis) derived by appropriately integrating the basis functions of the *M*-spline basis (see Ramsay, 1988). A quadratic monotone spline with a single interior knot $t \in [L,\, U]$ and intercept $\mu$ is of the form

6 $$\begin{aligned} f(x) = \mu + \sum _{i=1}^{3} \alpha _{i} M_{i} (x \mid t). \end{aligned}$$

In the proposed model, $t = L + 0.5(U - L)$ is chosen to lie halfway between the lower and upper limits $L$ and $U$, respectively. Monotonicity requires that $\alpha _{i} \ge 0$ for $i = 1,2,3$. The basis functions $M_{1}, M_{2}$ and $M_{3}$ are constructed to ensure continuity and first-order differentiability at $t$, and their formulae are as follows (Ramsay, 1988):

7 $$\begin{aligned} M_{1} (x \mid t)&= {\left\{ \begin{array}{ll}\nonumber \frac{2t(x-L)-(x^{2} - L^{2})}{(t - L)^{2}}, &{}\text {if}\quad L \le x < t;\\ 1, &{}\text {if}\quad t \le x \le U; \end{array}\right. }\\ M_{2} (x \mid t)&= {\left\{ \begin{array}{ll} \frac{(x-L)^{2}}{(t - L)(U-L)}, &{}\text {if}\quad L \le x < t;\\ \frac{t - L}{U - L} + \frac{2U(x - t) - (x^{2} - t^{2})}{(U - t)(U-L)} , &{}\text {if}\quad t \le x \le U;\\ \end{array}\right. }\\ M_{3} (x \mid t)&= {\left\{ \begin{array}{ll}\nonumber 0, &{}\text {if}\,L \le x < t;\\ \frac{(x-t)^{2}}{(U - t)^{2}}, &{}\text {if}\,t \le x < U;\\ \end{array}\right. } \end{aligned}$$

Hence (6) is simply a linear combination of these three piecewise quadratic functions with an intercept.

The spline functions are built into the column scores $\mathbf {b}$ in (5) by using the $(q-1) \times 4$ design matrix $\mathbf {M}$ to collect the basis functions corresponding to $\mu , \alpha _{1}, \alpha _{2}$ and $\alpha _{3}$, respectively. The basis functions are evaluated at the midpoints between rating categories, for example at 1.5, 2.5 up to 6.5 for a 7-point Likert scale. Hence $\mathbf {b}$ can be written as

8 $$\begin{aligned} \mathbf {b} = \begin{pmatrix} \mathbf {b}_{1} \\ \mathbf {b}_{2} \\ \end{pmatrix} = \begin{pmatrix} \mathbf {b}_{1} \\ \mathbf {M} \varvec{\alpha } \\ \end{pmatrix} \end{aligned}$$

with $\mathbf {b}_{1}$ the $m$-vector of unrestricted object scores and $\mathbf {b}_{2}$ the $(q-1)$-vector of spline-restricted boundary scores. The spline parameters are collected in $\varvec{\alpha } = (\mu , \alpha _{1}, \alpha _{2}, \alpha _{3})^{'}$.

**Fig. 2:**
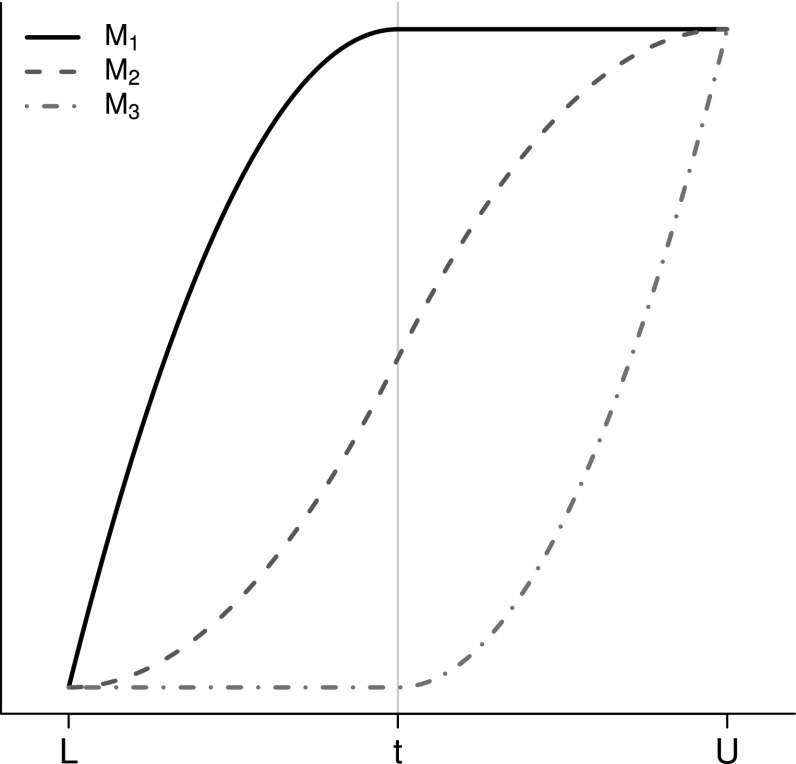
The three *I*-spline basis functions for quadratic monotone splines with a single interior knot $t$.

The basis functions $M_{1}, M_{2}$ and $M_{3}$ in (7), as depicted in Figure 2, are piecewise quadratic, with only two of them nonconstant in each of the intervals $[L,\, t)$ and $[t,\, U]$. This is convenient because it means the second derivative of $f$, and hence the curvature, depends only on two parameters in each interval. Rescaling without loss of generality so that $L = 0$ and $U = 1$, the curvature of $f$ (not necessarily defined at $t = 1/2$) is given by

9 $$\begin{aligned} \frac{d^{2}}{dx^{2}} f(x) = {\left\{ \begin{array}{ll} - 8 \alpha _{1} + 4\alpha _{2}, &{}\text {if}\quad 0 \le x < 1/2;\\ - 4\alpha _{2} + 8\alpha _{3}, &{}\text {if}\quad 1/2 < x \le 1;\\ \end{array}\right. } \end{aligned}$$

The function $f(x)$ is either convex, concave or linear in a given interval depending on whether the second derivative (9) is positive, negative or zero, respectively, which does not depend on $x$. In fact, assuming that $\alpha _{1}$ and $\alpha _{3}$ are larger than zero, the curvature can be measured solely in terms of the ratios $\alpha _{2}/\alpha _{1}$ and $\alpha _{2}/\alpha _{3}$, referred to henceforth as the curvature parameters. For example, the requirement for convexity in both the lower and upper domain is

10 $$\begin{aligned} \frac{d^{2}}{dx^{2}} f(x) > 0 \Leftrightarrow {\left\{ \begin{array}{ll} \frac{\alpha _{2}}{\alpha _{1}} > 2, &{}\text {if}\quad L \le x < t;\\ \frac{\alpha _{2}}{\alpha _{3}} < 2, &{}\text {if}\quad t < x < U.\\ \end{array}\right. } \end{aligned}$$

When one or both of $\alpha _{1}$ and $\alpha _{3}$ are zero, one or both of these curvature parameters may be undefined. This can cause problems for its graphical representation, some of which will be shown below. In such cases, a continuity adjustment through the addition of a small positive constant to both the numerator and denominator in (10) can be useful.

**Fig. 3:**
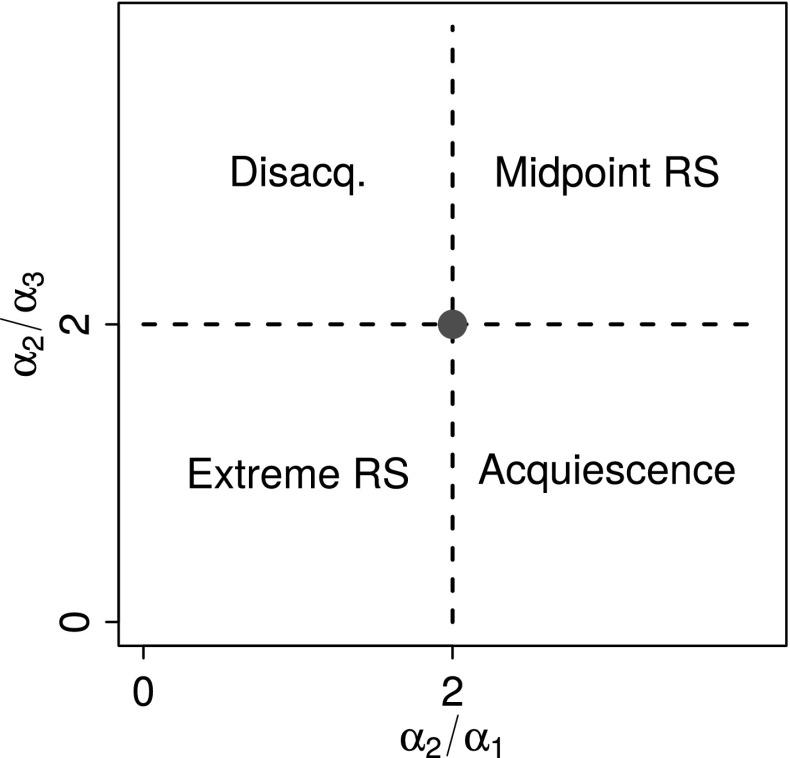
Classifying response styles graphically using the curvature properties of monotone quadratic splines.

It is possible to rewrite Table 1 wholly in terms of the curvature parameters, but more importantly using the curvature parameters it is possible to visualize the curvature of an estimated response style in a single plot. Figure 3 illustrates the situation by plotting $\alpha _{2}/\alpha _{3}$ against $\alpha _{2}/\alpha _{1}$, as well as incorporating the response style classification regions derived from Table 1. When both curvature parameters equal two, no response style is present. Due to the fact that both curvature parameters has the range $[0, \infty )$, a more symmetric plot is arrived at by using the base-2 logarithmic transform and centring—this is illustrated in Section 5.

### Dual Scaling Method for Multiple Response Styles

To allow for multiple response styles, suppose that each of the $n$ individuals belongs to one of $K$ response style groups, the exact membership being unknown. Let the $n \times K$ matrix $\mathbf {G}$ contain as columns the group indicator vectors $\{\mathbf {g}_{k}\}_{k=1}^{K}$, each indicating which individuals belong to that specific group. The column scores $\{ \mathbf {b}_{k} \}_{k=1}^{K}$ are of the same form as $\mathbf {b}$ in Equation (8), but are now group-specific by replacing $\mathbf {b}_{2}$ with $\mathbf {b}_{2k} = \mathbf {M} \varvec{\alpha }_{k}$. This allows for the different groups to have different response mappings by letting the spline parameters $\varvec{\alpha }_{k} = (\mu _{k}, \alpha _{1k}, \alpha _{2k}, \alpha _{3k})^{'}$ vary between groups. The object scores $\mathbf {b}_{1}$ and the row scores $\mathbf {a}$ remain fixed across all response style groups.

The group membership $\mathbf {G}$ needs to be estimated, together with the $2n$-vector $\mathbf {a}$ of optimal scores for the individuals and the column score vectors $\mathbf {b}_{k}$ of length $(m+q-1)$ contained in the $K$ columns of $\mathbf {B}$. It is required for monotonicity that $\alpha _{ik} \ge 0$ for all $i$ and $k$. The loss function in Equation (5) must be adjusted to allow for the multiple response styles as well as for the spline restrictions. This constrained DS method for the detection of response styles can be formulated as

11 $$\begin{aligned}&\min _{\mathbf {a}, \mathbf {B}, \mathbf {G}} L ( \mathbf {a}, \mathbf {B}, \mathbf {G})\nonumber \nonumber \\&\text {subject to}\,\mathbf {b}_{k} = \begin{pmatrix} \mathbf {b}_{1}\\ \mathbf {b}_{2k} \end{pmatrix} \; \text {and}\;\alpha _{ik} \ge 0,\; i = 1, 2, 3,\; k = 1, 2, \ldots ,K. \end{aligned}$$

The adjusted loss function (compare Equation (5)) is

12 $$\begin{aligned} L ( \mathbf {a}, \mathbf {B}, \mathbf {G}) = c\Vert \mathbf {F}_{r} - \frac{1}{2} (m + q - 2) ( \mathbf {1} \mathbf {1}^{'} + \sum _{k=1}^{K} \mathbf {D}_{\mathbf {g}_{k}} \mathbf {a} \mathbf {b}_{k}^{'} ) \Vert ^{2}. \end{aligned}$$

Again, $c$ is a proportionality constant, and the diagonal matrices $\mathbf {D}_{\mathbf {g}_{k}}$ are constructed as

13 $$\begin{aligned} \mathbf {D}_{\mathbf {g}_{k}} = \begin{pmatrix} \mathrm {diag}{\left( \mathbf {g}_{k}\right) } &{} \mathbf {0} \\ \mathbf {0} &{} \mathrm {diag}{\left( \mathbf {g}_{k}\right) } \\ \end{pmatrix}. \end{aligned}$$

In this context, $\mathrm {diag}(\mathbf {x})$ denotes the diagonal matrix with $\mathbf {x}$ on the main diagonal. Through using the $\{\mathbf {D}_{\mathbf {g}_{k}}\}_{k=1}^{K}$ in (12), individuals are associated with the corresponding $\mathbf {b}_{k}$ for their group. As K increases, the number of parameters in the model increases and consequently the loss function $L$ decreases as well. Therefore, when considering how the value of $L$ changes for different values of $K$ in a scree plot, it is convenient to standardize these values to the unit interval $[0,1]$.

An algorithm for minimising $L$ is discussed in the next section.

### An Alternating Nonnegative Least Squares Algorithm

Solving the optimization problem in (11) requires finding $\mathbf {a}, \mathbf {B}$, and $\mathbf {G}$ under the appropriate restrictions. The approach discussed here alternates over two steps:

1. The algorithm combines ALS and nonnegative least squares (NNLS; Lawson and Hanson, 1974) to approximate the optimal $\mathbf {a}$ and $\mathbf {B}$ for a given group membership matrix $\mathbf {G}$. This involves fixing the value of $\mathbf {a}$, estimating the optimal $\mathbf {B}$ with NNLS, and then updating $\mathbf {a}$ by ordinary least squares (OLS) based on the estimate of $\mathbf {B}$. This ALS process is repeated for a given $\mathbf {G}$ until numerical convergence is observed.
2. For fixed $\mathbf {a}$ and $\mathbf {B}$, $\mathbf {G}$ is updated by a $K$-means type algorithm given the values determined for $\mathbf {a}$ and $\mathbf {B}$. This step simply allocates each individual sequentially to the group which minimises the loss function.

The algorithm alternates between steps one and two until the loss function $L$ changes by less than a small positive constant. Note that starting values for both $\mathbf {a}$ and $\mathbf {G}$ are required. For the $\mathbf {a}$ vector standard normal random numbers are simulated, while random assignment to $K$ groups is used for $\mathbf {G}$. Block-relaxation algorithms such as this is guaranteed to converge monotonically, albeit to a local minimum; therefore multiple random starts are required (De Leeuw, 1994). The related issues of local optima in $K$-means clustering and categorical principal components analysis are discussed in Hand and Krzanowski (2005) and Kooij (2007, Chapter 2), respectively. In Appendix 2 an overview of these local optima is given in the context of the empirical example (Section 5).

The optimization process is described in more detail in Algorithm 1, with an exposition of its derivation deferred to Appendix 1. The formulation is for a single starting configuration of $\mathbf {G}$, and needs to be repeated for multiple such configurations. Parameters that need to be specified include $n_{a}$, the number of (random) starts used for $\mathbf {a}$, the maximum number of iterations $\text {maxit}_{a}$ and $\text {maxit}_{G}$ for the ALS and $K$-means phases, respectively, and also the numerical tolerances $\epsilon _{1}>0$ and $\epsilon _{2} >0$ for these two steps. Note that the spline restrictions are sufficient as normalization constraints in the ALS part of the algorithm, and hence the vector $\mathbf {a}$ is only standardized to $\Vert \mathbf {a}\Vert ^{2} = 2n$ after convergence.

To update $\mathbf {G}$, the algorithm cycles through all respondents in turn. For the current respondent $i$, it calculates for each class what the loss function would be if respondent $i$ were assigned to that class, given the current classification of all other respondents. This respondent is then moved to the class with minimum loss (or stays in the same class if this is already the best choice). The algorithm then proceeds to the next respondent $i + 1$, and starts again with respondent 1 once the last respondent is reached. Once a complete pass over all respondents are made where no change in classification occurs, the updating of $\mathbf {G}$ terminates and the algorithm returns to the ALS updating step.
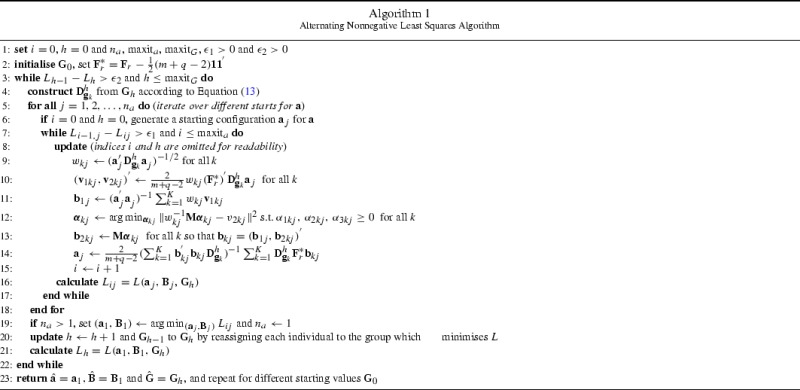


### Selecting the Number of Response Style Groups

To select the number of groups $K$, a scree plot of the loss function for different values of $K$ can be used. The aim is to choose the smallest $K$ such that larger values do not significantly reduce the loss. This method was introduced by Cattell (1966) and has been widely adopted. The DS method also separates individuals based on the shape of the response transformations and rating frequencies in the groups. This supplementary information can be helpful for choosing $K$ in cases where the scree plot is not conclusive. This will be illustrated in the empirical application of Section 5.

### Purging Response Styles

Once the estimates $\hat{\mathbf {a}}, \hat{\mathbf {B}}$ and $\hat{\mathbf {G}}$ have been obtained, these can be used to create a version of the original data $\mathbf {X}$ which is purged of response styles. All that is needed is to use the splines estimated for each response style group to assign optimal scores to the rating scale. Then these scores are substituted in $\mathbf {X}$ by replacing every rating with the appropriate optimal score.

Determining the optimal scores of the ratings proceeds by evaluating the splines as before, but now at the ratings themselves and not at the boundaries. This simply requires constructing a design matrix from the spline basis functions evaluated at the rating categories 1 to $q$, where for categories 1 and $q$, respectively, $L$ and $U$ are used in the notation of Section 3.2. As before, a single interior knot $t$ at the middle of the domain $[L, U]$ of the splines are assumed. Let this matrix be $\mathbf {M}^{*}$. The optimal scores are then simply determined as $\mathbf {M}^{*} \varvec{\alpha }_{k}$. In Section 4.3 a simulation experiment is conducted to assess how accurately this method can reproduce a known underlying correlation structure from contaminated data.

## Simulation Results

### Simulation Model

The simulated data was generated in a three-step procedure. First, the true underlying preference structure for the $m$ objects is obtained by simulating $m$ random numbers from a $U(0,1)$-distribution. These are gathered into the $m$-vector $\varvec{\mu }$. Second, individual preferences are generated by simulating $n$ times from each of $m$ truncated normal distributions respectively centred at the elements of $\varvec{\mu }$. The individual preferences are given by $\varvec{\delta }_{i} = \varvec{\mu }+ \varvec{\varepsilon }_{i}$, with $\varvec{\varepsilon }_{i}, i = 1, \dots , n$, representing the individuals deviation from the mean.

Truncation is done at 0 and 1 so that response styles can be defined on the closed interval $[0,1]$. Note that the use of truncation avoids overflow problems at the lower and upper ends of the response style mapping, and hence improves on the original approach of Van de Velden (2007). The truncated normal draws are done independently and with error variance $\sigma ^{2}$, which is an important parameter because it determines how pronounced the multi-modality of the mixture of truncated normals over $[0,1]$ is. An increase in the value of $\sigma$ implies that it easier to detect response styles as the actual preference structure plays less of a role in forming the ratings.

The resultant true preferences are randomly divided into different response style groups. Finally, these data are discretized to $m$ categorical variables with $q$-point Likert-scales, according to the cut points on $[0,1]$ implied by the chosen $K$ response styles. These response styles are parameterized to come from the family of monotone quadratic splines outlined in Section 3.2.

In the simulations, choices must be made regarding the following: the number of objects $m$, the number of rating categories $q$, the underlying standard deviation $\sigma$, the number of response styles $K$, as well as their shapes defined by $\varvec{\alpha }_{k}, k = 1, \ldots , K,$ the sample size $n$ and how this is divided among the $K$ groups, namely $n_{k}, k = 1, \ldots , K.$

### Assessing Classification Performance

The first simulation study assesses the classification accuracy of the DS method. It is assumed in this experiment that the number of groups $K$ is known beforehand. For each of the experimental conditions, 50 simulated data sets were constructed and the DS method applied. For each data set estimation was based on 15 random starts for $\mathbf {G}$, and for each of these starts the ALS procedure was initialised from 50 different random configurations for the row scores $\mathbf {a}$.

**Fig. 4:**
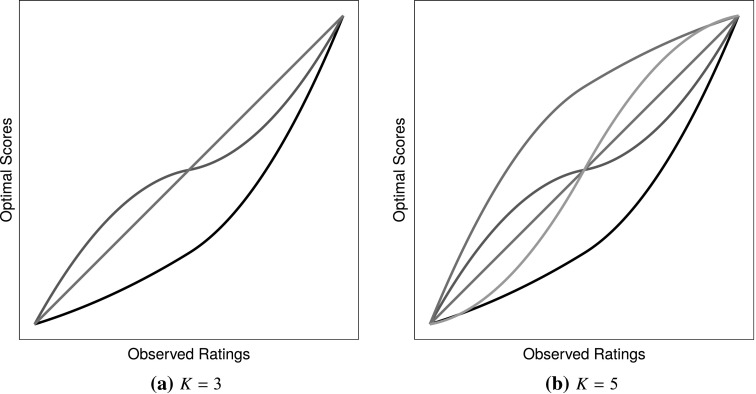
Response styles used in the simulation study. Each curve represents a different style.

The 108 experimental conditions consisted of the following. The number of objects $m$ was varied over 10, 20 and 30 items. The rating scales employed were either $q = 5$ or $7$-point scales. Sample sizes of $n = 200$, $1000$ and $5000$, respectively, were used. The number of groups $K$ were either 3 or 5. For each of these $K$, it was assumed that one of the groups has a linear response mapping (that is, a group with no response style). The additional $K-1$ groups exhibited response styles through nonlinear mappings. For $K = 3$, these additional groups were acquiescence and extreme responding, since Baumgartner and Steenkamp (2001) found that these are most prevalent in survey data. For $K = 5$, groups for disacquiescence and midpoint responding were also added. The corresponding spline functions used to simulate from are shown in Figure 4. The sample of $n$ respondents was assigned to the groups by allocating either 20, 50 or 80 % of respondents equally among the $K-1$ response style groups. These percentages represent the amount of contamination in the simulated data. The remaining percentage of respondents was assigned to the group exhibiting no response style. The latent standard deviation $\sigma$ was fixed at 0.1 for all experiments.

To assess the classification performance of the method, the adjusted Rand index as well as the percentage correctly classified (the so-called hit rate) were computed. The adjusted Rand index (ARI) of Hubert and Arabie (1985) assesses the similarity between two partitions, adjusted for chance correspondences between these partitions. The upper limit of the ARI is one, and indicates perfect agreement. An ARI of zero indicates that the method does not improve on random assignment, with all positive values indicating an improvement. Negative ARI values are also possible, and indicate poorer performance than random assignment. The ARI is in general lower than the hit rate, and can be considered as a more objective measure of performance.

For each of the 108 experimental conditions, Tables 2 and 3 show the average values over the 50 simulated data sets. It is apparent that the sample size $n$ does not have a large influence on the ARI and hit rate. The number of groups $K$ is very important for performance when the contamination percentage is low (20 %). This is because for $K = 5$ groups the 20 % of contaminated data points must be divided into four groups instead of 2 when $K = 3$, which results in groups with very low proportions $n_{k}/n$ of the total sample. The low performance here is somewhat compensated for by using more items, such as $m = 30$, but for $K = 5$ groups even more items are needed. In general, using more items increases the classification accuracy. Using a larger number of rating categories $q$ also increases performance, but mostly so with fewer groups ($K = 3$). The method improves on random assignment—especially in cases with higher response style prevalence and 20 or more items the improvement is substantial.

**Table 2:** Average adjusted Rand index for 50 simulations at the different parameter settings.

	$q = 5$									$q = 7$								
	$n = 200$			$n = 1000$			$n = 5000$			$n = 200$			$n = 1000$			$n = 5000$		
RS%	$m=10$	20	30	10	20	30	10	20	30	10	20	30	10	20	30	10	20	30
$$K = 3$$																		
20	0.28	0.40	0.61	0.30	0.42	0.62	0.29	0.40	0.62	0.31	0.48	0.74	0.29	0.48	0.80	0.30	0.48	0.80
50	0.59	0.80	0.90	0.57	0.80	0.91	0.58	0.80	0.91	0.62	0.85	0.93	0.64	0.86	0.94	0.62	0.85	0.94
80	0.73	0.90	0.93	0.72	0.89	0.95	0.75	0.89	0.95	0.75	0.91	0.96	0.76	0.90	0.96	0.76	0.91	0.96
$$K = 5$$																		
20	0.16	0.22	0.33	0.16	0.22	0.34	0.16	0.21	0.34	0.17	0.24	0.35	0.17	0.25	0.36	0.18	0.25	0.36
50	0.42	0.65	0.82	0.42	0.65	0.81	0.42	0.65	0.82	0.44	0.67	0.86	0.44	0.66	0.84	0.44	0.66	0.85
80	0.70	0.85	0.93	0.70	0.86	0.93	0.71	0.86	0.93	0.73	0.88	0.94	0.73	0.88	0.95	0.73	0.88	0.95

**Table 3:** Average hit rates for 50 simulations at the different parameter settings.

	$q = 5$									$q = 7$								
	$n = 200$			$n = 1000$			$n = 5000$			$n = 200$			$n = 1000$			$n = 5000$		
RS%	$m=10$	20	30	10	20	30	10	20	30	10	20	30	10	20	30	10	20	30
$$K=3$$																		
20	0.66	0.76	0.87	0.67	0.77	0.87	0.67	0.76	0.88	0.69	0.81	0.92	0.67	0.81	0.94	0.68	0.81	0.94
50	0.84	0.93	0.97	0.83	0.93	0.97	0.84	0.93	0.97	0.85	0.95	0.98	0.86	0.95	0.98	0.86	0.95	0.98
80	0.87	0.96	0.97	0.87	0.95	0.98	0.89	0.95	0.98	0.88	0.96	0.98	0.89	0.96	0.99	0.89	0.96	0.98
$$K=5$$																		
20	0.50	0.56	0.68	0.50	0.57	0.70	0.49	0.56	0.69	0.51	0.60	0.70	0.50	0.61	0.71	0.52	0.61	0.72
50	0.72	0.86	0.93	0.71	0.86	0.93	0.71	0.86	0.93	0.73	0.87	0.95	0.74	0.87	0.94	0.74	0.87	0.94
80	0.84	0.93	0.97	0.84	0.94	0.97	0.85	0.94	0.97	0.86	0.95	0.98	0.86	0.95	0.98	0.86	0.95	0.98

### Recovering Underlying Structure through Data Cleaning

**Fig. 5:**
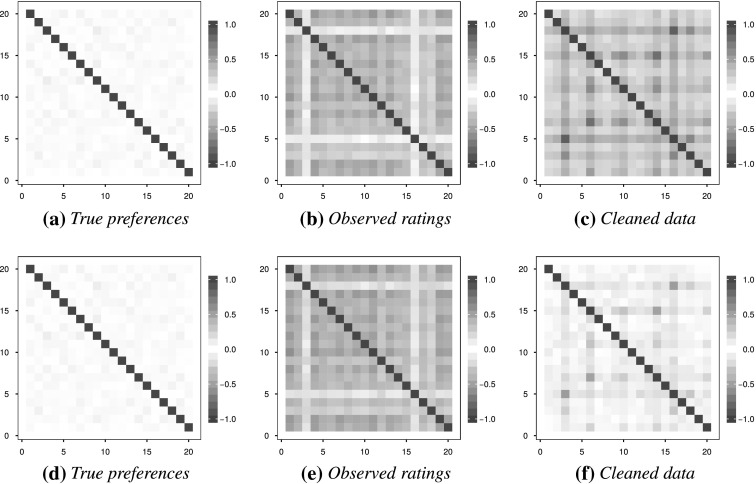
The effect of response styles on the underlying uncorrelated objects: estimated Pearson correlations before and after contamination, as well as after cleaning the data. The number of rating categories is $q=5$ for (**a**)–(**c**) and $q=7$ for (**d**)–(**f**), with $m=20$ items in all cases.

The simulation model of Section 4.1 assumes that, given the expected value of the object scores $m$, the objects are independently distributed as truncated normal distributions. Although the true correlation matrix between the objects thus is the identity matrix $\mathbf {I}$, the observed correlations after the response style contamination is often inflated. To show improvement, the cleaned data derived as in Section 3.6 should have correlations resembling independence more closely. A visual example is given in Figure 5 for simulated data ($m = 20, K = 3$ similar to the conditions used in Tables 2 and 3), where the colours indicate the magnitude of the Pearson correlations. It is evident that the response styles artificially inflate the correlations. When $q=7$, the cleaned data to some extent succeeds in removing the spurious correlations, but when $q=5$ the situation is not much improved.

The conditions under which the cleaned data can be expected to provide a better representation of the underlying correlation matrix was studied further through simulations. For the different values of $K, n, q,$ and the proportion of response style contamination used in Section 4.2, 50 simulated data sets were constructed and cleaned through the DS method. Here $m=20$ was fixed for simplicity. For each of these data sets, the root mean square error (RMSE) between $\mathbf {I}$ and the empirical Pearson correlation matrix for the contaminated data was calculated, where

14 $$\begin{aligned} \mathrm {RMSE}(\mathbf {V},\mathbf {W}) = \sqrt{\sum _i\sum _j (v_{ij} - w_{ij})^2} \end{aligned}$$

for commensurable matrices $\mathbf {V}$ and $\mathbf {W}$. Similarly, the RMSE comparing $\mathbf {I}$ with the empirical Pearson correlations of the cleaned data can be computed. A reduction in the RMSE when using the cleaned data as opposed to the contaminated data indicates that the cleaned data has a correlation structure which matches the true correlation structure more closely.

A two-sample Wilcoxon test, also known as the Mann–Whitney test, (e.g. Rice, 2007) was used to test the null hypothesis that the RMSE is equal for the contaminated and cleaned data against the one-sided alternative that the RMSE for the contaminated data is greater than that of the cleaned data. The results are quite clear: when $q=7$ the null hypothesis is always rejected ($p < 0.001$) in favour of the alternative, whilst when $q=5$ the null hypothesis cannot be rejected even once (all $p > 0.2$). It can therefore be deduced that when a sufficient number of rating categories $q$ are used, the correlation structure of the cleaned data is more representative of the true underlying structure of the data.

A related question concerns the performance of the method in the presence of a nontrivial correlation structure. To impose such a structure whilst retaining truncated normal marginal distributions for the objects, a copula is used (note that the truncated multivariate normal distribution does not guarantee truncated normal marginals). A copula is a multivariate distribution function $C(u_1,u_2,\ldots ,u_m)$ with uniform marginals (Hofert & Mächler, 2011). According to Sklar’s theorem (Sklar, 1959; Hofert & Mächler, 2011) a multivariate distribution function $F$ with marginals $\{F_{j}\}_{j=1}^{m}$ can be constructed as

15 $$\begin{aligned} F(x_1,x_2,\ldots ,x_m) = C(F_1(x_1),F_2(x_2),\ldots ,F_m(x_m)). \end{aligned}$$

**Fig. 6:**
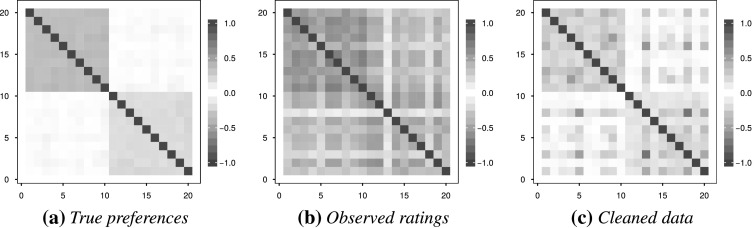
An example of the correlation structure imposed by the Clayton copula’s, in terms of Kendall’s $\tau$.

The marginal truncated normal distributions can be achieved by the inverse probability integral transform. The dependence structure between the variables is solely determined by the copula. Here two independent Clayton copula (Clayton, 1978) functions will be used to impose a correlation structure in terms of Kendall’s $\tau$, a well-known measure of rank correlation (see Kendall, 1938; Hofert & Mächler, 2011). The structure induced here for $m=20$ is as follows: the first ten objects are correlated with $\tau = 0.2$, independent of the other ten objects which are correlated with $\tau = 0.35$. These $\tau$ values amount to Pearson correlations of approximately $\rho = 0.3$ and $\rho = 0.5$, respectively (an approximate relationship is $\rho \approx \sin (\tau \pi /2)$—see Kendall and Gibbons (1990)). It is also possible to introduce negative correlations by using $1-U$ instead of $U$ in the inverse probability integral transform. In the application here these reversals are made randomly with differing probability $\gamma$. The theoretical, observed and cleaned correlations given by Kendall’s $\tau$ for one such copula is illustrated in Figure 6, with $m=20$ and $q=7$.

The difference in RMSE can again be used to evaluate the effect of the data cleaning on the correlation structure, now using Kendall’s $\tau$ since the Clayton copula’s use this measure directly. A simulation study was conducted for $m=20$ objects with the other parameters varying as before. For each combination of the parameters, the RMSE was calculated for 50 randomly generated data sets according to the copula model described above. Then for each data set the constrained DS model was fit as before, and a cleaned data set constructed. The difference in the RMSE for the contaminated data as compared to the cleaned data was recorded.

Table 4 presents the average reduction in RMSE as a result of cleaning the data with the DS procedure. As before the two-sample Wilcoxon test was performed. Significant improvements were found in all cases except those printed in italic in Table 4. It is apparent that the cleaned data improves the RMSE in all cases, except where both $q$ and $K$ are small and the proportion of contamination is moderate (50 %) to large (80 %). Except for these circumstances, the constrained DS method improves the estimation of the true correlation structure by removing the response styles effects.

**Table 4:** Average proportional improvement in the RMSE of the cleaned over the contaminated data.

	$q = 5$									$q = 7$								
	$n = 200$			$n = 1000$			$n = 5000$			$n = 200$			$n = 1000$			$n = 5000$		
RS%	$\gamma =0.5$	0.75	1.0	0.5	0.75	1.0	0.5	0.75	1.0	0.5	0.75	1.0	0.5	0.75	1.0	0.5	0.75	1.0
$$K=3$$																		
20	0.08	0.09	0.33	0.05	0.03	0.35	*0.02*	0.01	0.36	0.67	0.71	0.45	0.63	0.77	0.44	0.69	0.74	0.48
50	$-$ *0.10*	$-$ *0.09*	$-$ *0.07*	$-$ *0.09*	$-$ *0.14*	$-$ *0.24*	$-$ *0.07*	$-$ *0.02*	$-$ *0.15*	0.64	0.70	0.83	0.70	0.70	0.86	0.64	0.69	0.87
80	$-$ *0.41*	$-$ *0.37*	$-$ *0.44*	$-$ *0.34*	$-$ *0.41*	$-$ *0.47*	$-$ *0.38*	$-$ *0.43*	$-$ *0.46*	0.60	0.65	0.81	0.64	0.66	0.79	0.61	0.66	0.8
$$K=5$$																		
20	0.09	0.19	0.50	0.14	0.19	0.55	0.14	0.15	0.54	0.75	0.85	0.47	0.70	0.82	0.48	0.70	0.79	0.49
50	0.12	0.15	0.18	0.12	0.14	0.21	0.13	0.14	0.26	0.71	0.75	0.93	0.70	0.76	0.94	0.70	0.76	0.92
80	0.10	0.12	0.07	0.07	0.11	0.12	0.08	0.11	0.10	0.70	0.72	0.85	0.68	0.72	0.85	0.68	0.72	0.85

A two-sample Wilcoxon test for no difference in RMSE against the alternative hypothesis that the cleaned data significantly reduces the RMSE shows significant improvements ($\alpha = .95$) for all tests *except* those shown in italic print.

### Recovering the Parameters in Principal Components Analysis

It is possible to examine how well the method can recover parameters after the contaminated data have been cleaned of response styles. For simplicity, PCA (e.g. Johnson & Wichern, 2002) was used as analysis method, a well-known multivariate dimension reduction technique that seeks to summarize the majority of the variation in the data by a few uncorrelated linear combinations of the original variables (the so-called principal components). Subsequent principal components each account for as much variation in the data as possible, subject to being uncorrelated with the previous components. PCA relies on the eigendecomposition of the covariance (or correlation) matrix, where the eigenvalue-eigenvector pairs give the variance accounted for and the linear combination (also known as the principal component loadings), respectively, for each component.

The following procedure was used to compare the PCA conducted on the true correlation matrix to those conducted on the correlation matrices of the cleaned and contaminated data, respectively. First, a matrix of standard normal random numbers of dimension $m \times r$ is simulated, with $r$ denoting the required rank of the PCA solution. The rows are then standardized to length one; denote this matrix by $\mathbf {L}$. The simulated correlation matrix is then $\mathbf {R} = \mathbf {LL}'$, with the corresponding covariance matrix assumed to be $\mathbf {\Sigma } = \sigma ^{2} \mathbf {R}$. Here $\sigma ^{2}$ is the same error variance as assumed in Section 4.1. Since the decomposition $\mathbf {R} = \mathbf {LL}'$ is not unique, the eigendecomposition of $\mathbf {R}$ is used to re-express $\mathbf {R}$ as $\mathbf {R} = \mathbf {L}_{r}^{} \mathbf {L}_{r}'$, where $\mathbf {L}_{r}$ is constructed from the first $r$ eigenvectors and singular values of $\mathbf {R}$.

Second, a population mean vector $\varvec{\mu }$ for the $m$ items is simulated as uniform random numbers. The true underlying data for the respective respondents are then simulated from the multivariate normal distribution with mean vector $\varvec{\mu }$ and covariance matrix $\mathbf {\Sigma }$. The resultant matrix represents the uncontaminated data. Subsequently, response styles are added to arrive at the contaminated data. The same response styles as in Section 4.2 were used, the only difference being that the range $[L,U]$ of the splines was set to be the 1st and 99th percentiles of the sampled values, respectively. Any spillovers outside the range of the splines are then added to the lowest or highest rating category. The interior knot $t$ was fixed at the mean of the sampled values.

Finally, the constrained DS method was applied to the contaminated data, assuming that the correct number of response styles $K$ are known and using 15 and 50 random starts for $\mathbf {G}$ and $\mathbf {a}$, respectively. Based on this, a cleaned data set was constructed, from which the cleaned empirical correlation matrix, $\hat{\mathbf {R}}_{c}$ is obtained. Similarly, let $\hat{\mathbf {R}}_{o}$ be the empirical correlation matrix of the observed (i.e. the contaminated data). To compare the PCA solutions on these correlation matrices to that of $\mathbf {R}$, the decompositions $\hat{\mathbf {R}}_{c} \approx \mathbf {L}_{c} ^{}\mathbf {L}_{c}^{'}$ and $\hat{\mathbf {R}}_{o} \approx \mathbf {L}_{o}^{} \mathbf {L}_{o}^{'}$ are constructed as before assuming that the researcher is able to identify the correct rank $r$ of $\mathbf {R}$. The RMSE between $\mathbf {L}_{r}$ and $\mathbf {L}_{c}$ is then compared to that between $\mathbf {L}_{r}$ and $\mathbf {L}_{o}$ to determine whether the PCA structure of the cleaned data reflect the actual structure better or worse than the contaminated data.

For this simulation study, it was assumed that all groups are of equal size. The total sample size was varied over $n = 200$, 1000 and 5000 respondents as before, with either $K=3$ or 5 response styles added. Again, either $q=5$ or 7 response categories were studied, with $m = 10$, 20 or 30 items. The rank of $\mathbf {\Sigma }$ was either $r = 2, 3$ or 4. For each combination of these factors, 100 simulated data sets were analysed.

The results are shown in Table 5, which displays the average relative improvement in the RMSE of the cleaned over the contaminated data. It is evident that the PCA structure is better reflected by the cleaned data when $q = 7$. From the table it can therefore be concluded that rating scales of more than five categories are ideal for the method. For rating scales with $q = 5$, marginal improvements are seen only for small numbers of items. It is reassuring that the method does not yield significantly worse result for less refined rating scales such as $q = 5$. The improvement of the method is greatest for small values of $m$. The number of segments $K$ does not influence performance. Finally, the method performs best for low values of $r$, which corresponds to simpler underlying structures.

**Table 5:** Average proportional improvement in the RMSE when comparing the principal component loadings between the cleaned and contaminated data.

	$q = 5$									$q = 7$								
	$n = 200$			$n = 1000$			$n = 5000$			$n = 200$			$n = 1000$			$n = 5000$		
r	$m=10$	20	30	10	20	30	10	20	30	10	20	30	10	20	30	10	20	30
$$K=3$$																		
2	0.13	$-$0.04	$-$0.05	0.11	$-$0.05	$-$0.06	0.13	$-$0.03	$-$0.04	0.61	0.59	0.42	0.60	0.63	0.44	0.60	0.62	0.43
3	0.06	$-$0.03	$-$0.05	0.08	$-$0.03	$-$0.04	0.07	$-$0.02	$-$0.04	0.54	0.41	0.17	0.59	0.46	0.18	0.60	0.46	0.20
4	0.04	$-$0.02	$-$0.03	0.06	$-$0.01	$-$0.03	0.04	$-$0.03	$-$0.03	0.50	0.27	0.09	0.50	0.32	0.11	0.53	0.31	0.09
$$K=5$$																		
2	0.10	$-$0.00	$-$0.02	0.13	0.00	$-$0.02	0.10	0.00	$-$0.02	0.64	0.50	0.20	0.73	0.54	0.21	0.66	0.53	0.20
3	0.08	0.01	0.00	0.08	0.02	0.00	0.09	0.01	$-$0.00	0.61	0.30	0.11	0.63	0.28	0.10	0.66	0.32	0.08
4	0.06	0.03	0.01	0.07	0.03	0.02	0.08	0.03	0.01	0.55	0.19	0.10	0.60	0.24	0.10	0.61	0.21	0.09

## Application

To illustrate the method in an empirical application, consider data obtained from an anonymous multinational food and beverage conglomerate regarding an investigation of product perceptions for 20 similar products. These include in-house products as well as those of competitors. Data were collected from $n = 268$ panellists, who scored each product on seven different sensory attributes using a 9-point Likert scale. Each product is rated on all seven attributes (or, equivalently, items), so that there are 140 items collected in a data matrix with 268 rows and $m=140$ columns. The Likert scale ranges from 1 (“low”) to 9 (“high”), and hence $q = 9$. Since these products are generally liked by consumers, acquiescence can be expected. The data set is available in coded form as part of the **cds** package (Schoonees, 2015) for the statistical computing environment R (R Core Team, 2014). This can be obtained online from the Comprehensive R Archive Network. The package contains the software used for all computations in the present paper.

The first step is to select $K$ by inspecting the loss function through a scree plot. Consideration is also given to the curvature properties of the splines as well as how well the method separates groups of panellists who exhibit different distributions of rating scale use. It is expected that once spurious clusters are added at least two of the estimated response curves will be very similar, and/or that two groups will on aggregate use the rating scale in a very similar fashion. For each of $K = 1, 2, \ldots , 8$ groups, the algorithm was run from 50 different random starts for the grouping matrix $\mathbf {G}$, where appropriate. Also, 50 random starts for the ALS part of the algorithm was used. Appendix 2 gives insight into the effect of local optima for these data.

**Fig. 7:**
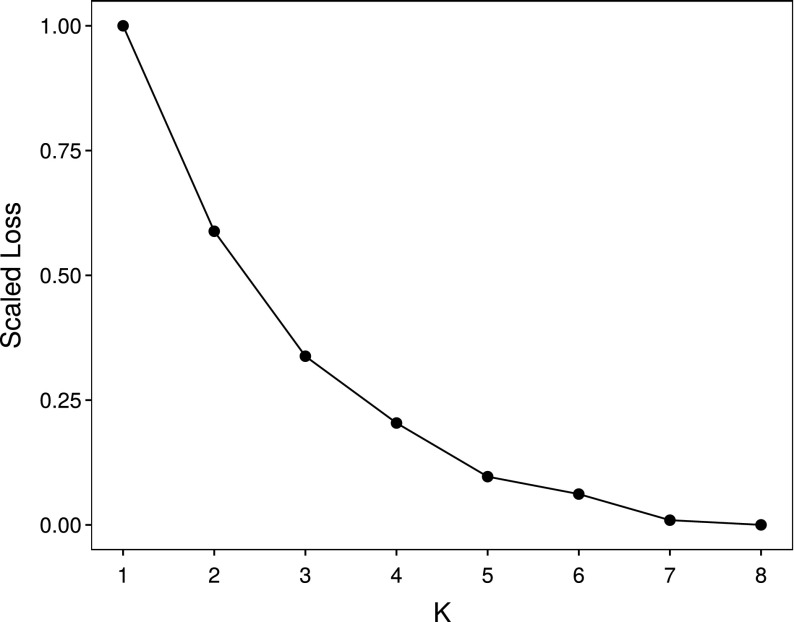
Scree plot for the sensory data.

Figure 7 shows the resulting (rescaled) scree plot. There does not seem to be a clear “elbow” in the plot, although it is apparent that $K = 3, 4$ and $5$ are the options requiring closer scrutiny. As $K$ increases beyond $5$ not much improvement in the loss function is observed.

**Fig. 8:**
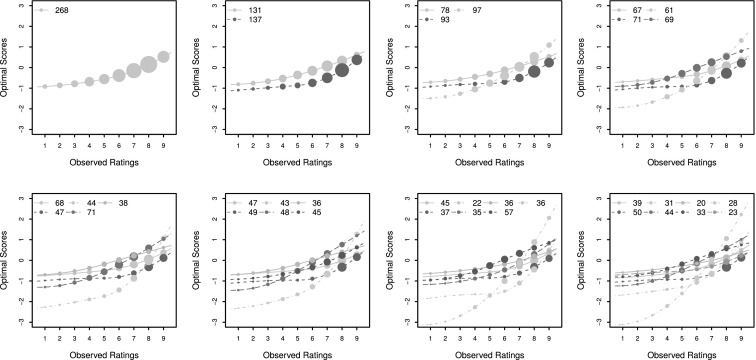
The estimated response mappings for $K = 1$ (*top left*) to 8 (*bottom right*) groups, respectively. The area of the bubbles are proportional to how often that particular rating is used. The group sizes are also shown in a legend. Groups are labelled sequentially; the legend should be read vertically and then horizontally.

The response mappings for the solutions $K = 1, \ldots , 8$ are displayed in Figure 8. In these plots the horizontal axis contains the original rating scale, while the vertical axis denotes the optimal scores assigned to the Likert scale. The area of the bubbles superimposed on the transformation plots indicate how often each rating category is used, aiding in the interpretation. A first observation is that (strictly, almost) all the detected response mappings have the characteristic convex shape of acquiescence. This means that all panellists have a tendency to use positive ratings frequently. The groups differ with respect to the intensity of the acquiescence.

Furthermore, the range of optimal scores that is assigned to each group, namely $\sum _{i=1}^{3} \alpha _{ik}$ in terms of the spline parameters set out in Sections 3.2 and 3.3, depends on the within-group variability of rating scale use. Groups where individual panellists’ rating scale use show more variability from the group’s aggregate rating scale use are assigned optimal scores with a wider range. Hence the method treats such groups, i.e. groups containing more individualistic respondents, as more informative as opposed to groups with more uniform response behaviour.

**Fig. 9:**
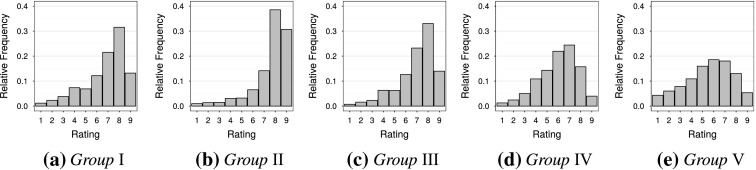
Relative aggregate frequencies of rating scale use in the identified groups when $K = 5$.

A closer look at the distribution of the rating scale use in the identified groups reveal that all groups in the solutions $K = 3, 4$ and $5$ show visually different distributions, except group I and group III when $K = 5$. The relative frequencies with which each rating is used in each of the groups when $K = 5$ are shown in the barplots in Figure 9. It is obvious that groups I and III have very similar aggregate behaviour when $K = 5$ . This is however not immediately apparent from the spline functions displayed in Figure 8, which assign different optimal scores to these groups.

A more formal comparison can also be made by using the Kullback–Leibler divergence (KL; e.g. Lehmann & Casella, 1998) between the distributions of different groups. This is also known as entropy distance and is often employed in the construction of classification trees (e.g. Breiman, Friedman, Stone, & Olshen, 1984). It is an asymmetric measure of the dissimilarity between two density functions, the reference density $f$ and another density $g$, which is defined as $E_{f} [\log (f(X)/g(X))]$. When $f = g$, the entropy is zero; otherwise it is positive. For discrete distributions the integral is replaced by a summation. In the present context, let $\hat{f}_{1}, \ldots , \hat{f}_{q}$ and $\hat{g}_{1}, \ldots , \hat{g}_{q}$ denote the observed proportion of all answers in two different groups that use ratings $1, \ldots , q$, respectively. The observed KL divergence between these groups, with respect to $\hat{f}$, is then $\sum _{h = 1}^{q} \hat{f}_{h} \log (\hat{f}_{h} / \hat{g}_{h})$.

Assessing the pairwise KL divergence for all pairs of groups (and using both $f$ and $g$ as reference) show that indeed the abovementioned two groups diverge the least among all pairs when $K = 5$—see Table 6. Since the method is designed to detect groups with different aggregate rating scale use it can be concluded that the addition of a fifth group is spurious and therefore $K=4$ is selected. The findings of Figure 9 are therefore supported by this analysis.

**Table 6:** The Kullback–Leibler divergence between the groups when $K = 5$, based on the rating scale use per group.

Group	I	II	III	IV	V
I	–	0.158	0.009	0.187	0.234
II	0.161	–	0.138	0.699	0.701
III	0.008	0.134	–	0.224	0.297
IV	0.166	0.606	0.202	–	0.053
V	0.231	0.680	0.317	0.065	–

The distributions of the groups in the rows are treated as the respective reference distributions, $f$.

**Fig. 10:**
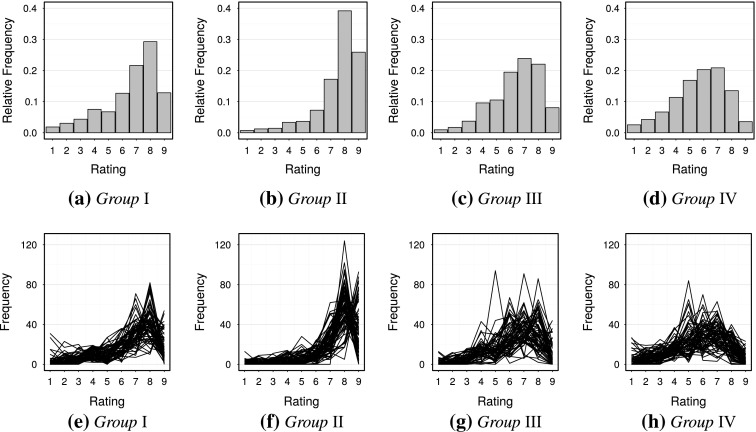
**a**–**d** Relative frequencies of rating scale use for the chosen solution $K=4$; and **e**–**h** Variability of rating scale use within these groups, with each line representing a single individual.

**Fig. 11:**
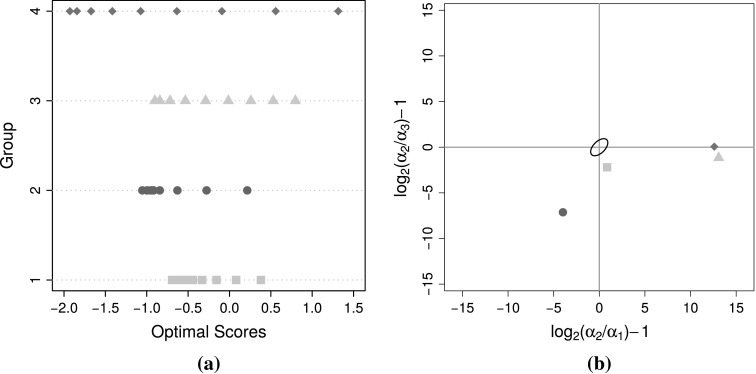
**a** Optimal scores assigned to the $K = 4$ response style groups, from rating 1 (*left*) to rating 9 (*right*). **b** Curvature plot similar to Figure 3 for the four groups, with the axes now transformed to obtain a more symmetrical plot. The ellipse in the centre is an approximate 95 % confidence ellipse for no response style.

Consider the results for $K = 4$ groups. These four groups consist of 67, 71, 61 and 69 panellists, respectively. The rating scale usage of these groups are displayed in Figure 10, panels (a)–(d). Figure 11 displays the optimal scores assigned to the ratings in the different groups as well as their curvature chart. The curvature chart includes an approximate 95 % confidence ellipse constructed for the parameter estimates of 5000 data sets simulated under the assumption that no response styles exist. Any group falling outside this band therefore has a significantly nonlinear response mapping and hence a response style.

Group I represents acquiescence as mainly ratings 6–9 are used by panellists. There is a slight boundary effect, as also with the other groups, in that category 9 is used less often than category 8. Because the ratings 6–9 are frequently used, the optimal scores assigned to these are close to zero. The most meaningful optimal scores are assigned to the lower categories since when these are used it contains more information for this group of panellists. Overall the information provided by this group is low since the range of optimal scores assigned is very narrow. This is because the group members display low variability with respect to their rating scale use. This is evident from Figure 10(e), which plots the frequency with which each rating is used per individual. Group II represents a more extreme acquiescence where categories 7–9 are often used. The range of assigned optimal scores, and hence information, is similarly narrow, but shifted further to the left since the upper categories are used even more frequently. Since the response mapping is concave in the lower part of the domain there is a slight deviation from acquiescence towards an extreme response style.

Groups III and IV both exhibit a mix of acquiescence and midpoint responding. This is evident from the relative frequencies in Figure 10 and the curvature chart in Figure 11(b). In these groups generally ratings 4–8 are preferred. Based on the range of optimal scores assigned to them these consist of the panellists providing the most information. Especially Group III is endowed with the most meaningful spread of optimal scores, and can be seen in Figure 10(g) to exhibit the most within-group variation.

**Fig. 12:**
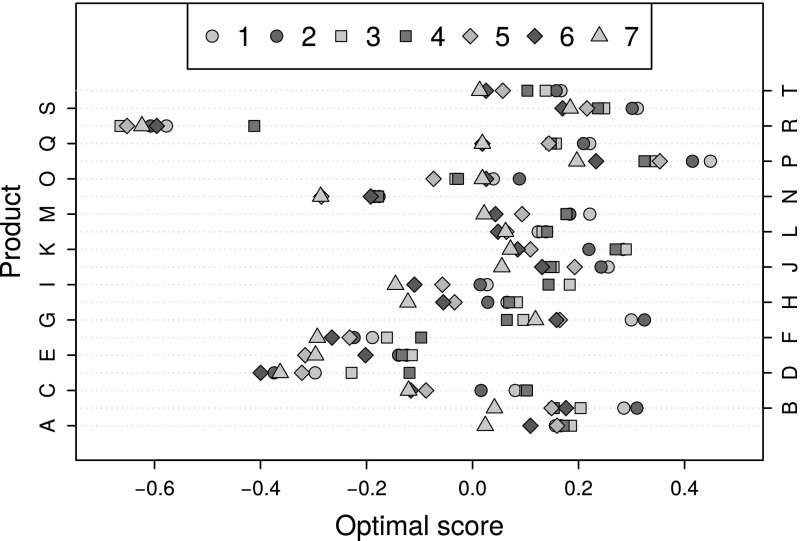
Optimal scores for each of the seven questions, separated by product and with similar items depicted by the same colours.

Finally, consider the optimal scores assigned to the items as displayed in Figure 12. It is evident that Product R, and to a lesser extent Products N, D, E and F, received the lowest ratings. In contrast, Product P was the best performing one. By using a cleaned data set constructed by replacing the ratings by optimal scores further analyses can be conducted which are less influenced by the presence of the response styles.

## Conclusions

A method that relies on the properties of DS for successive category data to detect response styles in categorical data was presented. It combines newly suggested spline models for four main types of response styles with the original DS method to construct optimal scores for the boundaries between rating categories. These optimal scores are sensitive to the presence of response styles. The method was adapted to allow for multiple response style groups by utilizing a $k$-means type procedure, which is combined with a constrained ALS algorithm using NNLS to fit the model.

Both the ability of the method to detect response styles and the improvement in correlation structure that results from a cleaned data set where ratings are replaced by optimal scores were studied. It was found that using 30 or more items and a rating scale of 7 or more categories yields great improvements in the classification of individuals to different response style groups. When fewer rating categories are used other factors become important, such as the extent to which response styles are present in the data. Also, when using a 7-point scale or more, the resulting cleaned data provide a more accurate description of the true substantial content in the data, after accounting for different response styles. The use of the method to identify respondents who provide similar amounts of information in their responses to a survey was illustrated on an empirical data set.

The number of response style groups to retain was selected on the grounds of a scree plot of the loss function, combined with the distribution of rating scale use in the different response style groups. It remains to be seen whether a more formal selection method can be derived. Other grounds for further research include alternatives for or additional restriction to the spline functions, and whether more freedom is needed by allowing for differences between the $m$ object scores in different groups.

## Figures and Tables

**Fig. 13:**
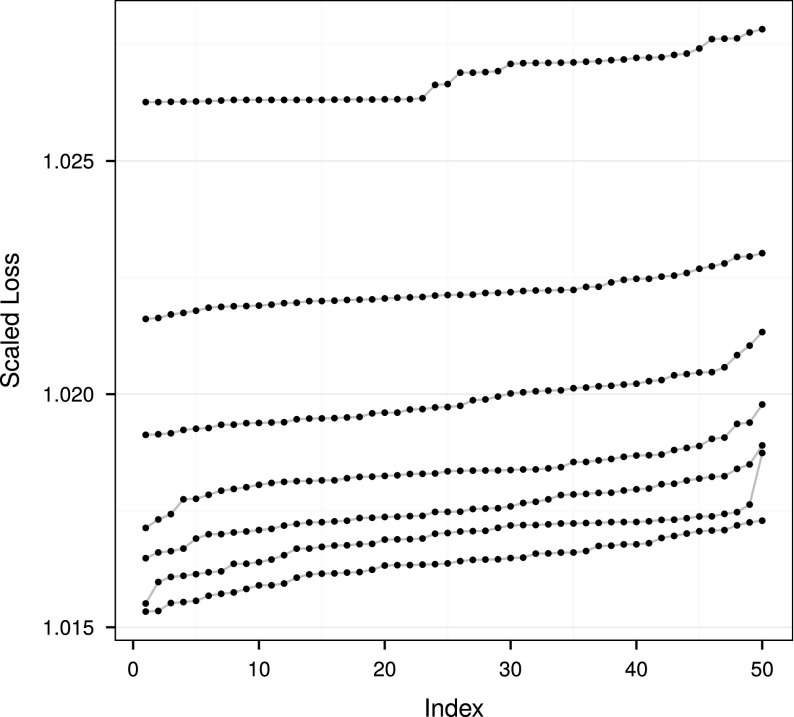
The spread of the loss values (scaled by a constant) for $K = 2, \ldots , 8$ in the empirical example, for 50 different starting configurations of $\mathbf {G}$.

## Appendix 1

Here an overview of the derivation of Algorithm 1 is provided (specifically, steps 9–14). Consider expanding the criterion of Equation (12), assuming without loss of generality that the proportionality constant $c = 1$:

16 $$\begin{aligned} L(\mathbf {a},\mathbf {B},\mathbf {G})&= \Vert \mathbf {F}_{r}^{*} - \frac{1}{2} (m + q - 2) \sum _{k=1}^{K} \mathbf {D}_{\mathbf {g}_{k}} \mathbf {a} \mathbf {b}_{k}^{'} \Vert ^{2} \nonumber \\&= \mathrm {tr\,}{\mathbf {F}_{r}^{*}}'\mathbf {F}_{r}^{*} + \frac{1}{4} (m + q - 2)^{2} \sum _{k=1}^{K} \mathbf {b}_{k}^{'} \mathbf {b}_{k}^{} \mathbf {a}^{'} \mathbf {D}_{\mathbf {g}_{k}} \mathbf {a} - (m + q - 2) \sum _{k=1}^{K} \mathbf {b}_{k}^{'}\mathbf {F}_{r}^{*'}\mathbf {D}_{\mathbf {g}_{k}}\mathbf {a}. \end{aligned}$$

This derivation uses $\mathbf {F}_{r}^{*} = \mathbf {F}_{r} - \frac{1}{2}(m+q-2)\mathbf {1}\mathbf {1}^{'}$, the fact that $\mathbf {D}_{\mathbf {g}_{k}}$ is idempotent and that $\mathbf {D}_{\mathbf {g}_{k}}\mathbf {D}_{\mathbf {g}_{l}} = \mathbf {0} \; \forall \; k \ne l$, as well as the properties of the matrix trace operator. Note that the first term does not depend on the model parameters and hence are not used in the optimization algorithm.

Now, consider optimizing $\mathbf {a}$ and $\mathbf {B}$ when $\mathbf {G}$ is fixed. It follows from Equation (16) that, given a starting configuration of $\mathbf {a}$, the relevant loss function to be minimized for finding a new $\mathbf {B}$ is proportional to

17 $$\begin{aligned} L(\mathbf {B}\,|\, \mathbf {a}, \mathbf {G})&= \sum _{k=1}^{K} \left[ \frac{1}{4}(m+q-2)^2 \mathbf {b}_{k}^{'} \mathbf {b}_{k}^{} \mathbf {a}^{'} \mathbf {D}_{\mathbf {g}_{k}} \mathbf {a} - (m + q - 2)\mathbf {b}_{k}^{'}\mathbf {F}_{r}^{*'}\mathbf {D}_{\mathbf {g}_{k}}\mathbf {a} \right] \nonumber \\&= \frac{1}{4}(m+q-2)^2 \sum _{k=1}^{K} \Vert (\mathbf {a}^{'} \mathbf {D}_{\mathbf {g}_{k}} \mathbf {a})^{1/2} \mathbf {b}_{k} - \frac{2}{m+q-2}(\mathbf {a}^{'} \mathbf {D}_{\mathbf {g}_{k}} \mathbf {a})^{-1/2} \mathbf {F}_{r}^{*'}\mathbf {D}_{\mathbf {g}_{k}}\mathbf {a} \Vert ^{2} + c_{1} \end{aligned}$$

where the constant $c_{1}$ depends only on $K$ and $\mathbf {F}_{r}^{*}$. Hence $\mathbf {B}$, and, more specifically, the parameters $\mathbf {b}_{1}$ and $\varvec{\alpha }_{k}, , k = 1, 2, \ldots , K,$ are updated by minimizing:

18 $$\begin{aligned} \sum _{k=1}^{K} \Vert (\mathbf {a}^{'} \mathbf {D}_{\mathbf {g}_{k}} \mathbf {a})^{1/2} \mathbf {b}_{k} - \frac{2}{m+q-2}(\mathbf {a}^{'} \mathbf {D}_{\mathbf {g}_{k}} \mathbf {a})^{-1/2} \mathbf {F}_{r}^{*'}\mathbf {D}_{\mathbf {g}_{k}}\mathbf {a} \Vert ^{2}. \end{aligned}$$

Now, recall that $\mathbf {b}_{k} = (\mathbf {b}_{1}^{'}, \mathbf {b}_{2k}^{'})^{'}$ with $\mathbf {b}_{2k} = \mathbf {M} \varvec{\alpha }_{k}$, so that the relevant parameters in the $\{\mathbf {b}_{k}\}_{k=1}^{K}$ is $\mathbf {b}_{1}$ and $\{\varvec{\alpha }_{k}\}_{k=1}^{K}$. These parameters must therefore be updated using the loss function in Equation (18). Let $w_{k} = (\mathbf {a}^{'} \mathbf {D}_{\mathbf {g}_{k}} \mathbf {a})^{-1/2}$ and $\frac{2}{m+q-2}w_{k} \mathbf {F}_{r}^{*'}\mathbf {D}_{\mathbf {g}_{k}}\mathbf {a} = (\mathbf {v}_{1k}^{'}, \mathbf {v}_{2k}^{'})^{'}$. Since

$$\begin{aligned} \Vert (\mathbf {x}_{1}^{'}, \mathbf {x}_{2}^{'})^{'} - (\mathbf {y}_{1}^{'}, \mathbf {y}_{2}^{'})^{'} \Vert ^{2} = \Vert \mathbf {x}_{1} - \mathbf {y}_{1}\Vert ^{2} + \Vert \mathbf {x}_{2} - \mathbf {y}_{2}\Vert ^{2}, \end{aligned}$$

it follows that

19 $$\begin{aligned} L(\mathbf {b}_{1}, \varvec{\alpha }_{1}, \ldots , \varvec{\alpha }_{K}\,|\, \mathbf {a}, \mathbf {G}) = \sum _{k=1}^{K} \Vert w_{k}^{-1} \mathbf {b}_{1} - \mathbf {v}_{1k}\Vert ^{2} + \sum _{k=1}^{K} \Vert w_{k}^{-1} \mathbf {M}\varvec{\alpha }_{k} - \mathbf {v}_{2k}\Vert ^{2}. \end{aligned}$$

Therefore $\mathbf {b}_{1}$ can be updated by minimizing the first summation in Equation (19) by OLS independently of $\{\varvec{\alpha }_{k}\}_{k=1}^{K}$. Since $\varvec{\alpha }_{ik} \ge 0$ for all $i$ and $k$, the latter vectors are updated for each $k$ by using NNLS to minimize each of the individual elements of the second summation.

## Appendix 2

Here a short exposition is given of the spread of local optima for the empirical example. Specifically, the variability of the loss function for the 50 random starts of $\mathbf {G}$ is shown in Figure 13. The curves are ordered from $K = 2$ at the top to $K = 8$ at the bottom. It is evident that only a single random start typically produces the best result. In general, the local optima is less stable for larger values of $K$, as can be expected. It is evident from this example that attention must be paid to the number of random starts used in empirical applications of such algorithms. These results suggest that the “best of 20 random starts” rule often favoured by practitioners of $K$-means clustering may not suffice (Hand and Krzanowski, 2005); a pragmatic approach is required.
